# The Holobiont/Hologenome Concept Series

**DOI:** 10.1128/mBio.00605-16

**Published:** 2016-04-14

**Authors:** R. John Collier, Arturo Casadevall

**Affiliations:** a Harvard Medical School, Department of Microbiology and Immunobiology, Boston, Massachusetts, USA; b Johns Hopkins Bloomberg School of Public Health, Baltimore, Maryland, USA

## EDITORIAL

Over the past 2 years, we have made a concerted effort to expand the number and scope of minireviews published in *mBio* in order to help readers keep track of developments across the breadth of microbiology. In our conversations exploring potential subjects for minireviews, a topic emerged—the holobiont/hologenome concept—that was relevant to so many areas and of such high current interest that we decided it might serve as the basis for a collection of minireviews. The ensemble of articles comprising the current series is the result. We are grateful to the many authors who contributed manuscripts and to Margaret McFall-Ngai, who wrote the excellent introduction to the collection. This *mBio* series will certainly elicit vigorous discussion of what has become a topic of some controversy, and we invite discussants to submit their views in letters to the editor in *mBio.* We also invite suggestions of other topics of broad interest that might serve as the basis for additional collections of minireviews in *mBio*.

